# Characterization of glucose transporter-targeted lipid nanoparticles for enhancing n-3 PUFA utilization in slow-growing Korat chickens

**DOI:** 10.1038/s41598-025-33149-6

**Published:** 2025-12-21

**Authors:** Piyaradtana Homyok, Anyanee Kamkaew, Teerapong Yata, Worapapar Treesuppharat, Apipu Ariyachayut, Elisabeth Baéza, Cécile Berri, Amonrat Molee, Wittawat Molee

**Affiliations:** 1 School of Animal Technology and Innovation, Institute of Agricultural Technology, Suranaree University of Technology, Nakhon Ratchasima, 30000 Thailand; 2 School of Chemistry, Institute of Science, Suranaree University of Technology, Nakhon Ratchasima, 30000 Thailand; 3 Biochemistry Unit, Department of Physiology, Faculty of Veterinary Science, Chulalongkorn University, Bangkok, 10330 Thailand; 4 Drug Discovery and Development Center, Thammasat University Research Unit in Mechanisms of Drug Action and Molecular Imaging, Thammasat University, Pathumthani, 12120 Thailand; 5 INRAE, Université de Tours, BOA, Nouzilly, 37380 France

**Keywords:** N-3 polyunsaturated fatty acids, Lipid-based nanoparticles, Nanoencapsulation, Targeted delivery, Physiochemical characteristics, In vitro storage stability, In vivo biodistribution, Slow-growing chickens, Fats, Fatty acids, Lipid peroxides, Oils, Nanoparticles, Nanoparticles, Transporters, Fatty acids, Lipid peroxides, Oils, Imaging techniques and agents

## Abstract

The aim of this research was to investigate the synthesis of suitable carriers of nanoparticles for improving the utilization of n-3 polyunsaturated fatty acids (n-3 PUFAs) source in chicken diets. Lipid nanoparticles were successfully prepared with two different n-3 oil sources, tuna and algal oils using hot and high-pressure homogenization methods. Four preparations were defined as follows: non-targeting lipid nanoparticles containing tuna oil (TO_NPs), non-targeting lipid nanoparticles containing algal oil (AO_NPs), targeting lipid nanoparticles containing tuna oil (TO_TNPs) and targeting lipid nanoparticles containing algal oil (AO_TNPs). A second study was conducted for the targeting procedure, the treatments as follows: Control, TO_NPs and TO_TNPs. Thirty-three slow-growing chickens were examined during the post-administration kinetic at 2, 4, 8, 12 and 24 h. The physicochemical characteristics of lipid nanoparticles, storage stability and in vivo biodistribution were evaluated. The results showed that the particle diameters of TO_NPs and AO_NPs were 223.7 and 294.4 nm, whereas the particle diameters of TO_TNPs and AO_TNPs were 134.7 and 184.0 nm, respectively. The polydispersity index (PDI) and zeta-potential of nanoparticles showed a good distribution and stability in colloid dispersions, respectively. Moreover, the nanoparticles of the TNPs groups were less susceptible to lipid oxidation than that of the NPs groups during a storage at 4 °C. Biodistribution analysis based on Nile Red intensity indicated superior cellular uptake of TNPs. Fatty-acid profiling further confirmed this enhanced delivery, with TNPs increasing EPA and DHA deposition in breast muscle by approximately 26% and 35%, respectively, at 24 h post-administration compared with NPs. These results demonstrate the effectiveness of targeted lipid-based nanoparticles in facilitating direct transport of fatty acids into skeletal muscle cells.

## Introduction

Oils containing high amounts of n-3 polyunsaturated fatty acids (n-3 PUFAs) are susceptible to oxidation process^1^. Many studies have investigated strategies to improve storage stability of these oil sources, and the encapsulation technology was the more efficient^2,3^. Another possibility is to use nanotechnology to improve the utilization of n-3 PUFAs oil source^4^. This technology has a high potential, and we investigated its use to enhance the accumulation of n-3 PUFAs in chicken muscles to produce functional meat.

Korat chicken, a slow-growing breed that is very popular in Thailand, has been developed as an alternative option catering to the preferences of Thai chicken producers^5^ and could be used to produce functional meat enriched with n-3 PUFAs. Previous studies showed that dietary 4% tuna oil supplementation increased n-3 PUFAs content reaching 19.03% of total fatty acids in Korat chicken meat^6^.

However, n-3 PUFAs sources are susceptible to lipid oxidation that can affect the feed quality^7^. Although encapsulation improves oxidative stability to some extent, oxidation of n-3 PUFAs can still occur during feed processing and storage. Therefore, more advanced approaches such as nanoencapsulation with stabilizing agents have been explored to provide better protection against lipid degradation^8^. Beyond protection from oxidation, effective nutritional strategies should also improve the bioavailability of these fatty acids at the intended tissue site. In this context, ligand-targeted lipid nanoparticles offer the potential for both protection and targeted delivery, which this study aimed to evaluate in chicken breast muscle.

Several studies have demonstrated that lipid nanoparticles can be effectively formulated using edible oils, including fish oil rich in EPA and DHA^8–10^. However, the storage stability of such nanoparticles depends strongly on physicochemical properties particularly particle size, dispersibility, and zeta potential as well as the intrinsic susceptibility of PUFA-rich oils to oxidative degradation^8^. In addition, plain lipid nanoparticles may be susceptible to degradation by digestive enzymes, limiting their effectiveness in delivering bioactive compounds^11^. Surface modification is therefore critical not only for improving colloidal stability but also for influencing biological performance. Conventional stabilizers such as polyethylene glycol (PEG) can protect nanoparticles from aggregation and enzymatic degradation^12,13^, although PEG itself does not confer any targeting capability. Beyond stability enhancement, the nanoparticle surface can be functionalized with ligands recognized by specific transporters or receptors to enable targeted delivery^14–17^. Such ligand-directed strategies improve specificity and delivery efficiency to particular tissues.

In poultry, however, nanodelivery approaches have focused almost exclusively on non-targeted systems such as nanoencapsulated oils, emulsions, and solid or nanostructured lipid carriers primarily to enhance oxidative stability or bioaccessibility rather than to exploit transporter-mediated uptake mechanisms^18,19^. As a result, transporter-based targeting concepts that are well established in mammals have not yet been applied in poultry.

In this context, glucose-based ligands are of particular interest because glucose-derived moieties can engage GLUT-associated uptake pathways in certain muscle cell models^20^, and sugar-modified nanoparticles have also shown improved intestinal absorption after oral gavage in mammalian models, supporting the relevance of glucose-derived surface chemistry for transporter-associated delivery^17^. Additional support comes from our previous work in slow-growing Korat chickens, where alkyl polyglucoside (APG)–modified lipid nanoparticles exhibited markedly greater intestinal uptake than unmodified nanoparticles in both Caco-2 cells and in vivo intestinal tissue, and this uptake was competitively inhibited by glucose, indicating involvement of a glucose-associated interaction mechanism^21^. However, that study examined only intestinal interactions and did not evaluate whether glucose-associated mechanisms could enhance the delivery of n-3 PUFAs to edible tissues. The findings motivate investigation of whether a glucose-derived ligand such as APG could facilitate nanoparticle uptake and enhance EPA and DHA deposition in chicken muscle. To our knowledge, this work represents the first application of a glucose-based, transporter-informed nanodelivery system for targeted n-3 PUFA enrichment in poultry meat.

Therefore, this study aimed to investigate the physicochemical characteristics of lipid nanoparticles, their susceptibility to lipid oxidation during storage, and their ability to deliver the bioactive compounds (n-3 PUFAs) into the muscles. In feeding animal applications, an essential consideration is that lipid nanoparticles must remain stable during passage through the digestive tract and subsequent cellular adsorption to ensure efficient transfer to the target organ. In this study, lipid nanoparticles were administered in their pure form via oral gavage rather than being incorporated into the feed. This approach was intentionally employed to directly evaluate nanoparticle integrity, stability and their proposed targeting behavior in vivo without the confounding effects of feed matrix interactions or processing conditions.

## Results

### Physicochemical characteristics of lipid-based nanoparticles containing n-3 PUFAs oils

The physical characterization of nanoparticles containing n-3 PUFA oils (Tuna oil, TO and Algal oil, AO) are presented on Table 1. The mean particle diameter of the NPs groups was higher than that of the TNPs groups (*P* ≤ 0.001) whereas TO in both nanoparticle forms had smaller particle diameters than AO (*P* ≤ 0.001). The TO_TNPs group had the highest PDI value (*P* < 0.001) and the NPs groups had higher zeta-potential values than the TNPs groups (*P* < 0.001) and the value was highest in AO_NPs group.

**Table 1:** Physical characteristics of n-3 PUFA oils within different types of lipid-nanoparticles^1^.

Treatment	Average diameter (nm)	Polydispersity index	Zeta-potential (mV)
TO_NPs	223.7 ± 1.01^c^	0.315 ± 0.014^b^	-42.6 ± 0.47^b^
AO_NPs	294.4 ± 1.83^d^	0.261 ± 0.007^a^	-34.2 ± 0.46^c^
TO_TNPs	134.7 ± 1.18^a^	0.365 ± 0.008^c^	-48.1 ± 0.68^a^
AO_TNPs	184.0 ± 1.21^b^	0.298 ± 0.010^ab^	-49.4 ± 0.67^a^
P-value	≤ 0.001	≤ 0.001	≤ 0.001

^1^ Two different n-3 PUFA oil sources (tuna or algal, TO or AO) within different types of lipids targeted (TNPs) or not (NPs) nanoparticles.

Data are presented as mean ± standard error (SE) (*n* = 3). ^a−d^ Values in the same column with different superscripts letters are significantly different (*P* < 0.05).

### Thermal and chemical properties of lipid-based nanoparticles

**Fig. 1:**
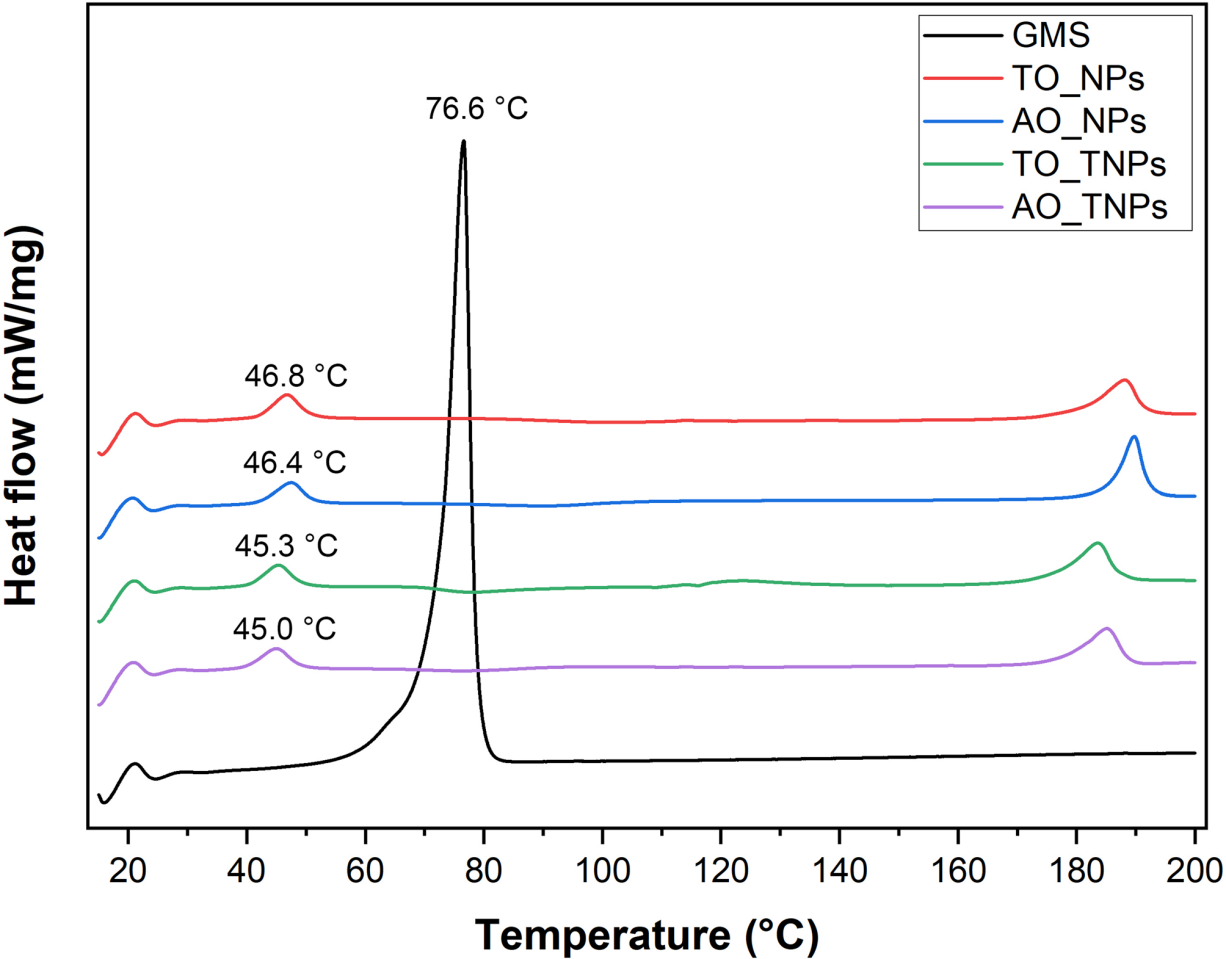
Differential scanning calorimetry (DSC) thermographs of nanomaterial including glyceryl monostearate (GMS), and lipid-based nanoparticles with tuna oil (TO) or algal oil (AO) and targeted (TNPs) or not (NPs).

The melting point of glyceryl monostearate (GMS), a raw material used for nanoparticle synthesis was at 76.6 °C, whereas the nanoparticle suspension including TO_NPs, AO_NPs, TO_TNPs and AO_TNPs showed lower endothermic peaks at 46.8, 46.4, 45.3 and 45.0 °C, respectively (Fig. 1). Both the n-3 PUFA sources and nanoparticle types changed the melting point in nanoparticle form by lipid nanoparticles composite of algal oil, and the targeting groups had the lowest melting points of lipid nanoparticles.

**Fig. 2:**
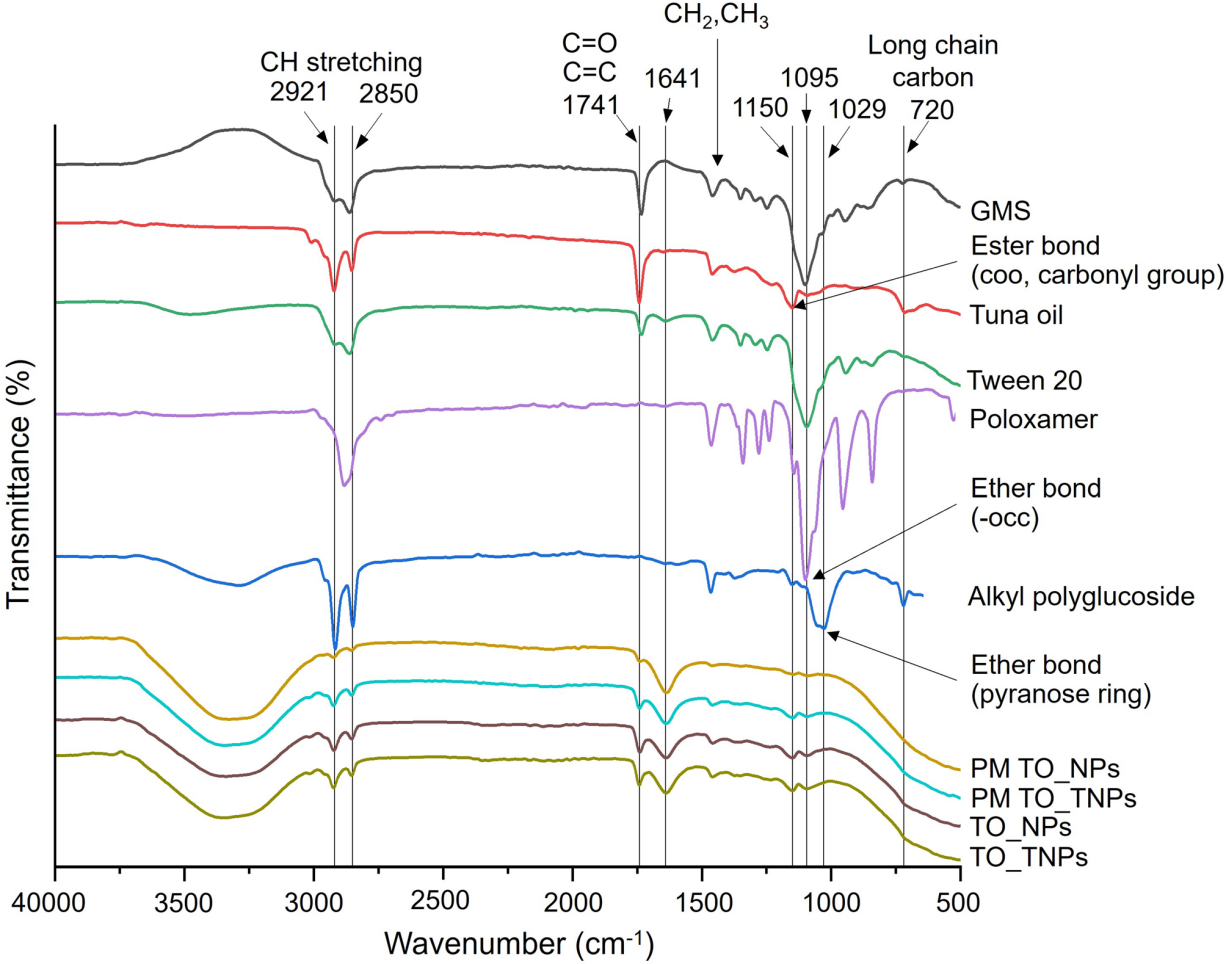
FTIR spectra of nanomaterials (GMS, tuna crude oil, tween 20, poloxamer, alkyl polyglucoside), physical mixture (PM) and lipid-based nanoparticles (NPs and TNPs).

The Fourier-transformed infrared spectroscopy (FTIR) analysis of nanomaterials and lipid-based nanoparticles is shown in Fig. 2. The peak intensity of lipid nanoparticles was more flattened than individual spectra of each material. It can be concluded that tuna crude oil was packed tightly inside the nanoparticles. Due to functional group which is composite in the structure of lipid, triglyceride and fatty acid that could be found in tween 20, alkyl polyglucoside, GMS and tuna oil, the peak intensity decreased after the synthesis into lipid-based nanoparticles.

The FTIR spectra shown in Fig. 2 illustrates the characteristic bands associated with hydroxyl groups (3499 –3233 cm^− 1^_,_ -OH stretching)^22^. was found in tween 20, poloxamer, alkyl polyglucoside and GMS. For lipid (2921 –2850 cm^− 1^, asymmetric and symmetric stretching vibration of CH_2_ groups in lipid alkyl chain, ν_asym_ CH_2_ and ν_sym_ CH_2_)^23^ and fatty acid (1641 cm^− 1^, C = C stretching, 1470–1460 cm^− 1^, -CH_3_ bending and 1240 –1207 cm^− 1^, -CH_2_ bending)^18,24,25^ were found in tween 20, alkyl polyglucoside, and tuna oil. The ester linkages (1741 –1734 cm^− 1^, ester carbonyl group (-C = O))^24^ was found in GMS, tween 20 and triglyceride in tuna oil but not occurred in alkyl polyglucoside and poloxamer. The cyclic ether (1029 cm^− 1^, cyclic ether (-COC) and pyranose ring (1150 cm^− 1^, -COC glycosidic bond)^26^ were found in alkyl polyglucoside. The wavelength at 720 cm^− 1^ indicated that this long chain carbon^27^ was found in GMS, tween 20, alkyl polyglucoside, and tuna oil. In addition, the wavelength ranging at 2921 –2850 cm^− 1^ (-CH stretching) and 1500 –500 cm^− 1^ (-CH bending and long chain carbon) had a reduced intensity in physical mixture and synthesized of lipid nanoparticles.

### In vitro storage stability of n-3 PUFA lipid-based nanoparticles

#### Particle size stability

The results of the storage stability on particle size of the n-3 PUFA lipid-based nanoparticles are shown in Table 2. The synthesis of n-3 PUFA source oils in the form of targeted-lipid nanoparticles groups (TO_TNPs and AO_TNPs) can help to maintain the size of the nanoparticles, but the storage conditions were better at low temperature storage (at 4 °C) for 4 weeks, while the particle size of the non-targeted lipid nanoparticles groups changed at a faster rate at all temperature storage (TO_NPs_4°C, TO_NPs_RT, AO_NPs_4°C and AO_NPs_RT).

However, during the storage, there were statistically significant changes in nanoparticle size for the AO_NPs_4°C (*P* < 0.05), AO_NPs_RT (*P* < 0.01), TO_TNPs_RT (*P* < 0.05), TO_TNPs_RT (*P* < 0.001) and AO_TNPs_RT (*P* < 0.001).

During the storage, the change in nanoparticle size was faster at room temperature than at 4 °C. In addition, the use of algal oil resulted in changing particle size faster in lipid-based nanoparticles (AO_NPs_4°C, AO_NPs_RT, and AO_TNPs_RT).

**Table 2:** Stability of n-3 PUFA oils within different types of lipid-nanoparticles^1^ on particle diameter during storage at room temperature (RT) or 4 °C.

Treatments	Particle diameter (nm)						
	Start	Week 1	Week 2	Week 3	Week 4	SEM	*P*-value
TO_NPs_4°C	225.23^cB^	215.83^cA^	233.37^cB^	197.03^bA^	230.02^cB^	4.09	< 0.01
TO_NPs_RT	223.50^cA^	233.83^dA^	258.83^cB^	258.8^cB^	268.88^dB^	6.02	< 0.05
AO_NPs_4°C	303.53^dAB^	328.93^eB^	316.87^dA^	300.4^dA^	320.03^eA^	3.62	< 0.05
AO_NPs_RT	299.60^dA^	332.17^eB^	341.83^dB^	358.53^eB^	344.78^fB^	5.93	< 0.01
TO_TNPs_4°C	136.50^a^	137.9^a^	139.67^a^	142.73^a^	139.4^a^	1.39	0.853
TO_TNPs_RT	134.00^aA^	137.47^aA^	131.23^aA^	136.5^aA^	140.82^aB^	1.04	< 0.05
AO_TNPs_4°C	176.7^b^	183.7^b^	177.57^b^	178.8^b^	183.51^b^	1.21	0.312
AO_TNPs_RT	176.4^bA^	187.53^bB^	194.00^bB^	192.13^bB^	195.09^bC^	1.92	< 0.001
SEM	12.92	14.89	15.40	15.43	15.23		
P-value	< 0.001	< 0.001	< 0.001	< 0.001	< 0.001		

^1^ Two different n-3 PUFA oil sources (tuna or algal, TO or AO) within different types of lipids targeted (TNPs) or not (NPs) nanoparticles.

Data are the average ± standard error of the mean (SEM) of three independent replicates (*n* = 3). For each attribute, different small letters indicate statistically significant (*P* < 0.05), highly significant differences (*P* < 0.01) and very highly significant differences (*P* < 0.001) between different samples at the same time of storage. Different capital letters in a same row indicate very highly significant differences (*P* < 0.001) over time.

#### Oxidative stability evaluated with peroxidative value

The peroxidative values are presented in Table 3. The unencapsulated tuna oil (TO_4°C) and tuna oil within lipid nanoparticles were stored at 4 °C (TO_NPs_4°C and TO_TNPs_4°C) and had reduced peroxidative values ​​(*P* < 0.001) compared to the groups kept at room temperature except at the start of the experiment where the values ​​were not different. Moreover, no statistically significant differences in peroxide production in unencapsulated algal oil were found between the two storage temperatures (AO_4°C and AO_RT) from week 1 to week 4.

In addition, algal oil within lipid nanoparticles (AO_NPs_4°C, AO_NPs_RT, AO_TNPs_4°C and AO_TNPs_RT at first day) showed a statistically highly significant (*P* < 0.001) increase in peroxide value after synthesis.

The synthesis of oil within nanoparticle form and storage at suitable temperature influenced the peroxidative value reaction. Pre-synthetic and post-synthetic oils in nanoparticle form kept at low temperature (at 4 °C) showed a statistically significant (*P* < 0.05) slowdown of peroxidative activity by comparison with the storage at room temperature from week 1 to week 3.

#### Oxidative stability evaluated with TBARS value

The malondialdehyde (MDA) values are presented in Table 4. Synthesizing tuna oil into the targeted-lipid nanoparticle increased the oxidation stability as the amount of MDA was lower in the non-targeted lipid nanoparticles but for algal oil, the reverse was observed. Moreover, storage at low temperature (at 4 °C) slows down the production rate of MDA. The synthesized algal oil within lipid nanoparticles in the experimental groups (AO_NPs_4°C, AO_NPs_RT, AO_TNPs_4°C and AO_TNPs_RT) had a greater increase in MDA than the unsynthesized algal oil (AO_4°C and AO_RT) (*P* < 0.001).

**Table 3:** Stability of n-3 PUFA oil sources within different type of lipid-nanoparticles^1^ on peroxidative value during storage at room temperature (RT) or 4 °C.

Treatment	Peroxidative value (meq of oxygen/kg fat)						
	Start	Week 1	Week 2	Week 3	Week 4	SEM	*P*-value
TO_4°C	0.106^dB^	0.110^cdB^	0.113^cdB^	0.079^cA^	0.184^cC^	0.009	< 0.001
TO_RT	0.113^dA^	0.160^fB^	0.213^iC^	0.153^eB^	0.306^dD^	0.018	< 0.001
AO_4°C	0.033^aC^	0.022^aA^	0.018^aA^	0.026^aB^	0.017^aA^	0.002	< 0.001
AO_RT	0.032^aA^	0.031^aA^	0.032^aA^	0.041^abB^	0.035^aAB^	0.001	< 0.001
TO_NPs_4°C	0.094^cAB^	0.108^cBC^	0.129^deD^	0.119^dCD^	0.085^bA^	0.005	< 0.001
TO_NPs_RT	0.095^cA^	0.130^deB^	0.196^hiC^	0.211^fC^	0.112^bAB^	0.013	< 0.001
AO_NPs_4°C	0.113^dA^	0.153^fB^	0.161^fgB^	0.164^eB^	0.161^cB^	0.006	< 0.001
AO_NPs_RT	0.111^dA^	0.210^gCD^	0.181^ghBC^	0.242^gD^	0.159^cB^	0.012	< 0.001
TO_TNPs_4°C	0.059^bB^	0.064^bB^	0.064^cB^	0.062^bcB^	0.039^aA^	0.003	< 0.001
TO_TNPs_RT	0.063^bA^	0.116^cdC^	0.097^cB^	0.107^dBC^	0.102^bBC^	0.005	< 0.001
AO_TNPs_4°C	0.107^dA^	0.099^cA^	0.127^dB^	0.108^dA^	0.103^bA^	0.003	< 0.001
AO_TNPs_RT	0.108^dA^	0.140^efB^	0.149^efBC^	0.164^eC^	0.114^bA^	0.006	< 0.001
SEM	0.005	0.009	0.01	0.011	0.013		
P-value	< 0.001	< 0.001	< 0.001	< 0.001	< 0.001		

^1^Two different n-3 oil sources (tuna or algal, TO or AO) within different types of lipid targeted (TNPs) or not (NPs) nanoparticles.

Data are the average ± standard error of the mean (SEM) of three independent replicates (*n* = 3). For each attribute, different small letters indicate very highly significant over time (*P* < 0.001) between different samples at a same time of storage. Different capital letters in a same row indicate highly significant differences over time (*P* < 0.001).

**Table 4:** Stability of n-3 PUFA oil sources within different type of lipid-nanoparticles^1^ on malondialdehyde value during storage at room temperature (RT) or 4 °C.

Treatment	Malondialdehyde value (µmol MDA/kg oil)						
	Start	Week 1	Week 2	Week 3	Week 4	SEM	*P*-value
TO_4°C	14.01^eC^	10.81^dB^	7.95^bcA^	14.89^dC^	13.32^deC^	0.69	< 0.001
TO_RT	13.76^eB^	14.08^efB^	17.97^fC^	6.53^cA^	17.9^fC^	1.13	< 0.001
AO_4°C	1.23^aB^	1.53^aC^	1.25^aB^	0.70^aA^	2.15^aD^	0.13	< 0.001
AO_RT	1.23^aA^	2.36^aC^	1.60^aB^	1.40^abAB^	2.82^aD^	0.17	< 0.001
TO_NPs_4°C	10.61^dA^	18.88^gC^	9.41^cA^	14.92^dB^	15.65^efB^	0.94	< 0.001
TO_NPs_RT	10.61^dA^	22.42^iD^	12.38^dB^	15.72^dC^	21.73^gD^	1.29	< 0.001
AO_NPs_4°C	9.21^cB^	15.38^fC^	9.10^cB^	6.75^cA^	8.51^cB^	0.79	< 0.001
AO_NPs_RT	8.88^cA^	21.06^hC^	21.65^gC^	20.75^eC^	13.55^deB^	1.39	< 0.001
TO_TNPs_4°C	13.70^eC^	13.03^eC^	6.42^bA^	8.58^cB^	9.07^cB^	0.76	< 0.001
TO_TNPs_RT	13.64^eA^	14.58^fA^	14.70^eA^	21.74^eB^	21.68^gB^	1.03	< 0.001
AO_TNPs_4°C	4.54^bB^	4.31^bB^	2.92^aA^	3.00^bA^	5.64^bC^	0.28	< 0.001
AO_TNPs_RT	4.54^bA^	7.89^cB^	8.15^cB^	7.61^cB^	11.4c^dC^	0.61	< 0.001
SEM	0.78	1.14	1.03	1.17	1.09		
P-value	< 0.001	< 0.001	< 0.001	< 0.001	< 0.001		

^1^ Two different n-3 oil sources (tuna or algal, TO or AO) within different types of lipids targeted (TNPs) or not (NPs) nanoparticles.

Data are the average ± standard error of the mean (SEM) of three independent replicates (*n* = 3). For each attribute, different small letters indicate very highly significant differences (*P* < 0.001) between different samples at the same time of storage. Different capital letters in the same row indicate very highly significant differences (*P* < 0.001) over time.

### Transfer of lipid nanoparticles to the target organ

**Fig. 3:**
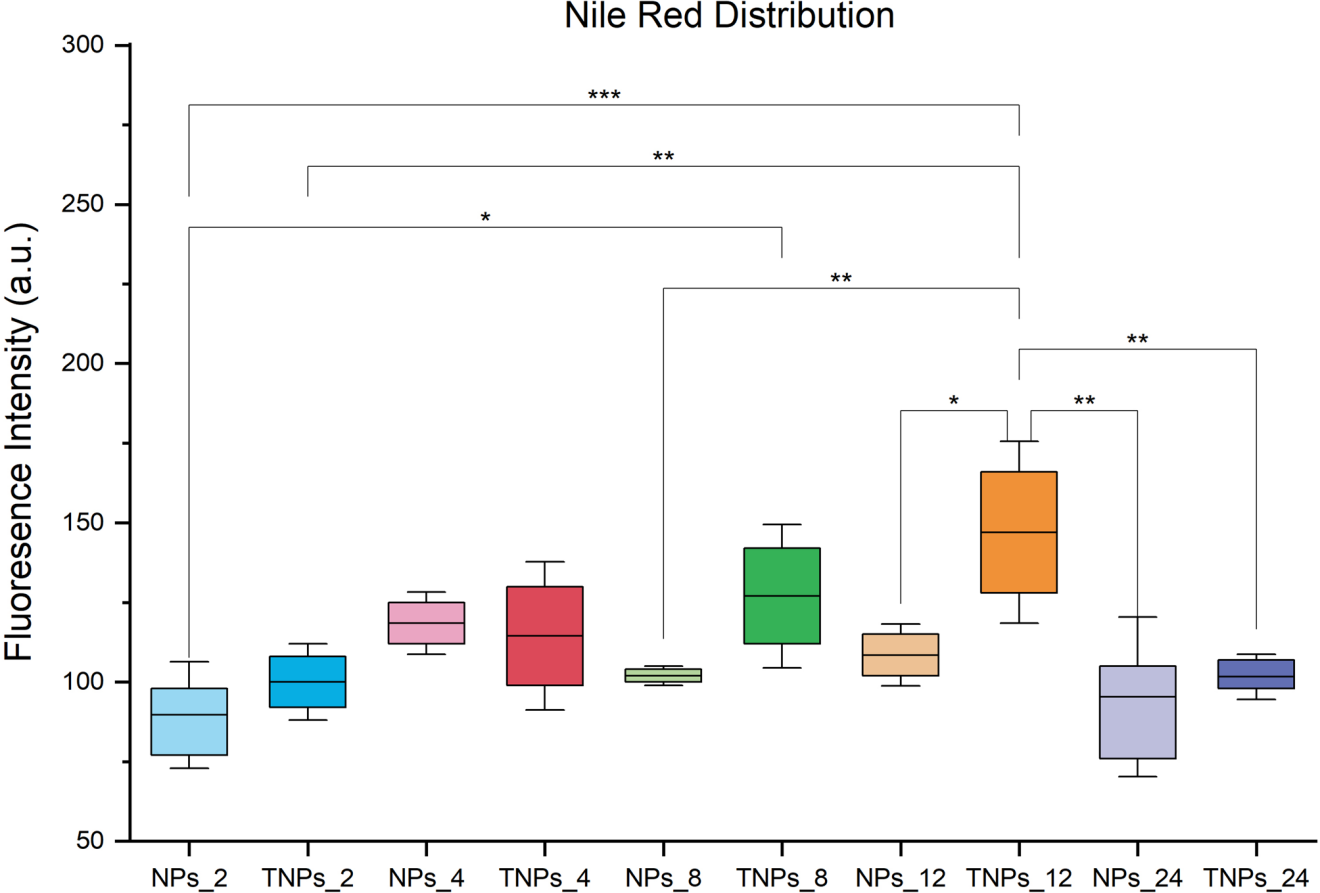
Nile Red distribution in Pectoralis major muscle of chicken imaging by IVIS of treatment group including non-targeted lipid nanoparticles (NPs) and targeted lipid nanoparticles (TNPs) at 2, 4, 8, 12 and 24 h after oral administration. The data are presented as bar graphs showing means ± SD (*n* = 3). *(*P* < 0.05), **(*P* < 0.01) and ***(*P* < 0.001).

The Nile red distribution in Pectoralis major sample is shown in Fig. 3. The Nile red distribution was increased within targeted-lipid nanoparticles (TNPs) at 8 and 12 h after oral gavage by comparison with non-targeted lipid nanoparticles (NPs). Then it decreased to the initial content at 24 h. The results demonstrated that the targeted lipid nanoparticles had the potential to be absorbed into the bloodstream and transferred to the target organ faster than other forms of nanoparticles.

#### Fatty acid composition of breast meat of Korat chicken

The fatty acid profile of breast meat of the chickens after oral gavage with the lipid-based nanoparticles is shown in Table 5. C18:3n3 content in breast meat of TNPs treatment at 4 h was decreased (*P* < 0.05) than that in the NPs treatment. NPs treatment at 4, 8 and 12 equal to control (*P* > 0.05) whereas TNPs treatment at all times point lower than control (*P* < 0.05). The EPA content in TNPs group at 4 h was higher (*P* < 0.05) than that in the NPs treatment. The DHA content in TNPs treatment at 4 h was higher (*P* < 0.05) than that in the NPs. By the way, the highest DHA content was shown in the TNPs group at 24 h. No significant difference in EPA contents at 2, 8, 12 and 24 h, and DHA content at 2, 8 and 12 h compared between NPs and TNPs treatment. C18:2n6 contents no different compared between NPs and TNPs at each time. C20:4n6 contents in TNPs treatment at 24 h higher (*P* < 0.05) than that in NPs treatment. But at 2, 4, 8 and 12 h showed no difference (*P* > 0.05). C18:1n9 contents in TNPs treatment at 24 h were lowest (*P* < 0.05) compared to that in NPs and control treatment.

#### Coefficient of efficacy of lipid-nanoparticles

The coefficient of efficacy of targeted and non-targeted lipid nanoparticles is shown in Table 6. TNPs had higher potential to transfer EPA and DHA into the chicken skeletal muscle than the NPs groups. The coefficient of efficacy of TNPs was higher than that of the NPs treatment in each time. At 2 h both NPs and TNPs treatments were able to transfer the n-3 PUFA but after that the TNPs treatment showed higher potential to transfer the n-3 PUFA at 4 and 24 h in TNPs treatment. The n-3 PUFA amount at 8 and 12 h after oral administration was decreased. In contrast, the n-3 PUFA content in NPs treatment was reduced at 4 h then increased slightly until 24 h.

**Table 5:** Fatty acid composition of TO oil within different types of lipid-nanoparticles^1^ at 2, 4, 8, 12 and 24 h after oral administration.

Treatment	Fatty acid composition (% of total fatty acids)							
	C16:0	C18:0	C18:1n9	C18:2n6	C18:3n3	C20:4n6	EPA	DHA
Control	19.52	9.31	20.33^b^	22.94^b^	0.35^d^	23.21^ab^	0.21^ab^	1.71^ab^
NPs at 2 h	18.62	9.13	20.23^b^	21.01^ab^	0.22^abc^	26.11^ab^	0.20^ab^	1.63^ab^
TNPs at 2 h	19.95	9.76	19.52^b^	21.69^b^	0.24^bc^	24.65^ab^	0.27^ab^	1.85^abc^
NPs at 4 h	20.57	9.84	21.75^b^	21.09^ab^	0.32^cd^	22.47^ab^	0.16^a^	1.57^a^
TNPs at 4 h	19.76	9.87	18.60^b^	20.18^ab^	0.20^ab^	26.60^ab^	0.28^b^	2.46^d^
NPs at 8 h	20.76	9.63	20.64^b^	22.35^b^	0.29^bcd^	22.02^ab^	0.21^ab^	1.82^abc^
TNPs at 8 h	19.91	8.96	20.16^b^	20.83^ab^	0.22^abc^	25.23^ab^	0.25^ab^	2.40^cd^
NPs at 12 h	20.18	8.55	25.89^bc^	22.13^b^	0.27^bcd^	18.75^a^	0.20^ab^	1.78^abc^
TNPs at 12 h	19.49	8.38	23.09^bc^	21.85^b^	0.23^abc^	22.38^ab^	0.23^ab^	2.22^bcd^
NPs at 24 h	18.62	9.24	19.75^b^	20.21^ab^	0.20^ab^	27.77^b^	0.23^ab^	2.55^d^
TNPs at 24 h	19.72	9.15	14.49^a^	17.50^a^	0.12^a^	35.62^c^	0.29^b^	3.43^e^
SEM	0.18	0.12	0.53	0.32	0.01	0.84	0.01	0.10
P-value	0.203	0.058	< 0.001	< 0.01	< 0.001	< 0.001	< 0.05	< 0.001

^1^ Tuna oil (TO) within different types of lipids targeted (TNPs) or not (NPs) nanoparticles.

Statistical analysis is based on F-test. Data are the average ± standard error of the mean (SEM) of three independent replicates (*n* = 3). For each attribute, different small letters indicate statistically significant (*P* < 0.05), highly significant differences (*P* < 0.01) and very highly significant differences (*P* < 0.001).

**Table 6:** Coefficient of transfer efficacy of TO within different type of lipid-nanoparticles^1^ at 2, 4, 8, 12 and 24 h after oral administration relative to control.

Treatment	Coefficient of efficacy (%)	
	EPA	DHA
Control	0.002	0.013
NPs at 2 h	0.62	0.76
TNPs at 2 h	2.32	2.27
NPs at 4 h	-1.41	-1.85
TNPs at 4 h	2.55	9.65
NPs at 8 h	0.06	0.64
TNPs at 8 h	0.80	4.93
NPs at 12 h	0.55	2.85
TNPs at 12 h	1.65	6.52
NPs at 24 h	1.07	9.70
TNPs at 24 h	2.38	16.98

^1^ Tuna oil (TO) within different types of lipids targeted (TNPs) or not (NPs) nanoparticles.

## Discussion

The in vivo study demonstrated that targeted lipid nanoparticles significantly improved the deposition of long-chain n-3 PUFAs in chicken breast muscle. These results highlight the effectiveness of the delivery system and suggest that nanoparticle composition and structure play key roles in bioavailability. To further understand these effects, omega-3 oil sources with different levels of bioactive compounds (EPA and DHA), along with the targeting process, were compared in terms of their influence on emulsion properties.

Physical characteristics such as particle size, PDI, and zeta potential differed clearly between TO- and AO-derived nanoparticles. These differences can be directly explained by the fatty acid composition, as shown in Table 7. AO contains a considerably higher proportion of long-chain PUFA, particularly DHA (37.63%), compared with TO (20.03%). Long-chain DHA has been reported to influence interfacial packing and increase droplet curvature, which is consistent with the larger particle sizes observed in AO nanoparticles^28^. In addition, AO nanoparticles exhibited a lower PDI than TO nanoparticles, indicating a more uniform size distribution. This outcome may be influenced by compositional factors inherent to the oil sources. The TO used in this study was a crude, unrefined tuna oil, which is typically reported to contain phospholipids and other minor components^29^ that can act as natural emulsifiers. Phospholipids have been shown to reduce droplet size and increase interfacial charge in lipid-based systems^30,31^, which may partly explain why TO nanoparticles displayed a smaller diameter and more negative zeta potential than AO nanoparticles. However, as the specific phospholipid content of the oils was not quantified in the present study, these interpretations should be considered as plausible mechanisms that warrant further investigation. In contrast, AO is a highly refined algal oil with fewer minor components, which likely contributed to its more uniform droplet formation and lower PDI.

The oxidative stability of the emulsions was influenced primarily by the intrinsic properties of the oils. Before nanoparticle synthesis, crude TO exhibited higher levels of lipid oxidation products than AO, indicating that TO enter the formulation process with a higher oxidative load due to its crude oil quality. This observation is consistent with reports showing that crude marine oils typically contain free fatty acids and other pro-oxidative constituents that increase susceptibility to oxidation^32^, whereas refined AO showed minimal initial oxidation in line with its higher purity and lower intrinsic oxidation rate^2,33,34^. After emulsification into nanoparticles, TO_NPs had lower peroxide values (PV) than AO_NPs, and TO_TNPs had lower PV than AO_TNPs. In contrast, the MDA content of TO_NPs and TO_TNPs was higher than that of AO_NPs and AO_TNPs, consistent with the role of MDA as a marker of secondary lipid oxidation^35^. These contrasting patterns likely reflect the distinct compositional profiles and oxidative histories of the two oils^36–38^. AO, with its higher PUFA content particularly DHA, which contains more double bonds and is highly reactive appears more prone to the rapid formation of primary oxidation products once dispersed as nanoscale droplets^37,38^. In contrast, crude TO had already accumulated higher levels of oxidation products before emulsification, so its greater MDA levels in nanoparticle form are consistent with a more advanced stage of lipid oxidation and a higher proportion of secondary oxidation products^35–37^. These contrasting oxidation behaviors suggest that DHA-rich refined algal oil and impurity-containing crude tuna oil may follow distinct oxidative pathways during and after nanoparticle formation. Future work should therefore include detailed profiling of oxidation intermediates, such as hydroperoxides, aldehydes, and volatile n-3 PUFA-derived oxidation products, to confirm these proposed mechanisms and more fully characterize the oxidative patterns of each oil. Based on these observations, tuna oil–loaded nanoparticles were selected for the in vivo experiment. This decision was informed not only by the relatively favorable oxidative behavior of TO-based nanoparticles under our synthesis conditions but also by the practical relevance of tuna oil in poultry production. As a widely available, cost-effective, and commonly used source of EPA and DHA in commercial diets, tuna oil provides a biologically meaningful and industry-relevant model lipid for evaluating targeted delivery efficiency in vivo.

The lower melting point of lipid nanoparticles mixtures than that of GMS due to the lipid matrix within the nanoparticles. Oils containing n-3 PUFAs (TO and AO) have liquid lipid properties leading to convert crystalline state to an amorphous state due to the disordered arrangement of molecules^8,9^.

Moreover, TO in lipid nanoparticles has a higher endothermic peak than algal oil due to undesirable compounds of tuna oil. Unrefined oil or triglyceride are more complex than free fatty acids and require more thermal energy^39^. Moreover, the melting point was affected by the high content in unsaturated fatty acids in the formulation^40^. The PUFAs implying a larger space to be accommodated into the polymeric matrix indicate induced a more amorphous structure of the lipid carrier^41^. Thus, there is reasonable that the endothermic peak in TO treatment was shifted at higher temperature than that AO treatment. The DSC is an effective tool to investigate the melting behavior and crystalline state of nanocarriers and their impact on some properties such as stability of lipid nanoparticles, which is useful for further use in animal feed processing.

During the storage at room temperature (RT), the diameter of nanoparticles was higher than that of nanoparticles stored at 4 °C particularly after 3 and 4 weeks of storage particularly for NPs treatment whereas there was no effect of temperature storage for TNPs treatment. The diameter of nanoparticles increased with storage duration. Similarly with literature research indicated the diameter of lipid nanoparticles was enlarged by storage for long time particularly unmodified-surface nanoparticles^30,42,43^. The reason is the aggregation of surfactants between nanoparticle^44^ or the interaction of reactive oxygen species (ROS) with surface of nanoparticle^45^. In contrast, the modified-surface nanoparticles can resolve the conflict and enhance oxidative stability by preventing pre-oxidation on the surface of the nanoparticle boundary^19,46^. Furthermore, the reduced particle size during storage due to the major compounds such as n-3 PUFAs in the matrix were descended by high temperature^46,47^.

The peroxidative value and the MDA level were higher during the storage at RT by comparison with the treatments stored at 4 °C. The high temperature can increase the kinetic energy contact of oil with oxygen^48^. The higher temperature had increased the rate of hydroperoxide decomposition. The reactivity of redox reactions had greater rates^49^. The amphiphilic lipid was reacted with the pro-oxidation at the interface and hydroperoxide was produced. The free radicals penetrate through the surface layer and stimulated the oxidation with n-3 PUFAs^50^. Thus, the study of storage stability with different temperatures indicated that lipid oxidation continuously occurred during storage particularly under high temperature for unmodified-surface nanoparticles. This should be noted that the storage of n-3 PUFAs in the lipid nanoparticle should be given priority for improving the sustainable quality prolonged enough for further procedures such as the drying process.

In addition, the targeting process influenced emulsion stability, as reflected by changes in polydispersity index (PDI) and zeta potential values. The PDI values describe the width or spread of the particles size distribution of the nanoparticles ranged between 0.1 and 0.3 indicating a good stability of the emulsion^51^. However, the TO_NPs group had the highest PDI value, possibly due to attractive hydrophobic interactions between the particles^52^. The use of liquid oils such as fish oil can make the nanoparticles more physically stable by reducing the crystalline structure. Therefore, it prevents the incorporation of nanoparticles^53^ and increases PDI value. Moreover, the lower PDI value in AO nanoparticles indicated that is more dispersion in suspension than that TO nanoparticles. The PD Index is based on size of nanoparticles^34^. The higher of the multiple particle size population shows more PDI value while higher uniformity is lower polydisperse result to lower the PD Index^54^.

Moreover, zeta-potential found in all groups was lower than − 30 mV. The NPs groups had lower values than the TNPs groups, (*P* < 0.001). The synthesis including tween 20, poloxamer and alkyl polyglucoside, resulted in a decrease in the electric potential difference of the nanoparticles^55^. For the NPs groups, the n-3 PUFA oils source influenced the zeta-potential value. This is due to the movement of carboxylic fatty acids in the region near the outer layer of the nanoparticles. This resulted in more negative charge conversion^28^. The TNPs groups contained alkyl polyglucoside. When this raw material is used in combination with a poloxamer, it results in a lower voltage difference than that observed for the NPs groups. Moreover, unrefined tuna oil contained phospholipids which are anionic groups^56^ and may have contributed to the high zeta-potential in TO_NPs group.

The melting temperature was higher for NPs nanoparticles than for TNPs nanoparticles because of targeting process effect on decreasing particle size. The size of particle became smaller resulting in decreased melting point^57^. By the way, the surface-modification of lipid nanoparticles could be effect on increase the particle size of nanoparticles^14,17^ whereas the particle size was decreased in the present study due to the ratio of targeting surfactant in formulation^58^.

It has been shown that the active substances and raw materials have similar properties, namely the lipid molecules, which affect the form of nanoparticles. The hydroxyl group (3499 –3233 cm^− 1^, -OH stretching) was found in raw materials with properties as an emulsifier, including tween 20^27^, poloxamer^59^, alkyl polyglucoside^60^ and GMS^32^. However, after the synthesis, the nanoparticles containing tuna oil had no effect on triglyceride degradation as it did not convert to free fatty acids, which should appear at wavelength of 1711 cm^− 1^^61^.

The wavelength at 1095 cm^− 1^ (-OCC, ether bond) provided by tween 20 and poloxamer implicated in the properties of emulsifier hydrophilic part of the molecule. The parts of raw materials, alkyl polyglucoside occur the wavelength at 1029 cm^− 1^ indicated the vibration of functional group of cyclic ether (-COC) and Pyranose ring and 1150 cm^− 1^ (-COC glycosidic bond)^26^.

The peak wavelength was changed towards a lower intensity in the physical mixture and lipid nanoparticles. It indicated that the raw material was integrated into the system before the nanoparticles would be formed. Moreover, the peak that disappeared at wavelength below 1095 cm^− 1^ in both mixtures was due to the structure of the molecules (including CH-stretching, long chain carbon) that bind together very densely. The nanomaterial changed their position from its own specific properties. The groups that had hydrophobic properties occurred together inside the particles, while the hydrophilic groups occurred around the periphery of the particles. This phenomenon presented the similar wavelength peak of -OH stretching a functional group found in the surfactant family of tween 20, alkyl polyglucoside, and poloxamer. Moreover, the apparent wavelength in the spectrum at 1741 –1734 cm^− 1^, 1641 cm^− 1^ and 1150 cm^− 1^ showed that the nanoparticles contained oil, such as tuna oil, inside the mixtures.

The wavelength ranging at 2921 –2850 cm^− 1^ (-CH stretching) and 1500 –500 cm^− 1^ (-CH bending and long chain carbon) disappeared or had a reduced intensity, indicating that the molecules in the mixture are moving according to the molecular properties mentioned above. These functional groups can be found in fat-containing raw materials, which have nonpolar molecules. This property is more prone to interactions together by noncovalent bonds such as van der Waals binding within particles^62^. Furthermore, the absence of new peaks in both mixtures showed that during the synthesis, there was no reaction that formed intermolecular covalent bonds^63,64^.

Although previous studies reported no temperature effect on nanoparticle size during storage^63,65,66^, the present study showed a clear temperature-dependent enlargement only in unmodified nanoparticles (NPs). This instability is likely driven by interactions with reactive oxygen species at the aqueous interface^54^ and by pro-oxidant impurities present in crude tuna oil^29,53^, which collectively enhances coalescence and oxidative degradation.

The storage behavior of TNPs differed markedly from that of NPs. Across all storage periods, TNPs showed minimal changes in particle size and oxidative parameters, whereas NPs exhibited progressive enlargement and greater MDA accumulation. This enhanced stability is attributed to the presence of alkyl polyglucoside (APG), a non-ionic surfactant that forms a protective interfacial layer around the lipid core. By providing steric stabilization and limiting the interaction of internal lipids with external oxidants, APG helps preserve the structural integrity of the nanoparticles. This mechanism aligns with previous studies demonstrating that surface-modified nanoparticles generally exhibit superior physical stability compared with unmodified systems^67–70^. This protective layer slows both hydrolysis and oxidation reactions, resulting in the minimal temperature-induced changes observed in TNPs.

Differences between oil sources also contributed to storage outcomes. Algal oil (AO) formulations changed more rapidly than tuna oil (TO), likely due to the higher proportion of free fatty acids in AO. Free fatty acids contain anionic carboxyl groups that migrate toward the particle surface and disrupt interfacial stability^61^. The exception was AO_TNPs stored at 4 °C, where low temperature slowed triglyceride hydrolysis and the release of DHA-rich free fatty acids^28^, resulting in smaller particle-size changes. These observations support that both oil composition and surface modification jointly determine physical stability.

In contrast, NPs lacking surface modification were more susceptible to structural and oxidative changes during storage. Hydrolysis of triglycerides can yield free fatty acids with negatively charged carboxyl groups, which tend to migrate toward the particle surface and promote oxidation^71^. This mechanism aligns with the higher MDA content observed in NPs. Furthermore, heating during nanoparticle synthesis may promote initial lipid oxidation, rendering NPs more vulnerable to temperature-driven degradation during storage.

Although PV values of all formulations remained below the general industry threshold for rancidity^72^, this threshold does not fully explain the divergent PV–MDA patterns observed in our nanoparticles. Lipid oxidation is inherently dynamic during storage, and hydroperoxides can rapidly progress to secondary oxidation products as the reaction continues^73^. It is therefore likely that hydroperoxides in AO- and TO-based nanoparticles were converted at different rates into downstream compounds, leading to low PV values despite active oxidation. This pattern is consistent with the elevated MDA levels observed in NPs. These results indicate that PV alone is insufficient to characterize oxidation kinetics in n-3 PUFA nanoemulsions. Future studies should therefore incorporate detailed profiling of hydroperoxides, aldehydes, and volatile oxidation markers to identify more robust indicators of oxidative stability across different oil sources.

The in vivo measurements of lipid-based nanoparticles demonstrated that the targeted formulation (TNPs) enhanced the deposition of n-3 PUFAs in skeletal muscle cells of Korat chickens. Nile red staining clearly showed that fatty acid accumulation in muscle at 8 h and 12 h post-gavage was higher in chickens receiving TNPs than in those receiving non-modified nanoparticles (NPs). These results indicate that APG-modified nanoparticles may interact more efficiently with intestinal epithelial surfaces, thereby promoting the delivery of fatty acids to systemic tissues.

Although the exact transport mechanism cannot be fully confirmed based on the present data, these observations are consistent with our previous findings using the same APG-LNP platform in Korat chickens^21^. In that study, glucose-modified nanoparticles exhibited faster and markedly higher uptake in Caco-2 intestinal cells than non-modified nanoparticles, and fluorescence accumulation in the ileum after oral administration was approximately 27-fold higher for APG-LNPs compared with LNPs. Importantly, uptake of APG-LNPs in Caco-2 cells was dose-dependently inhibited by both D- and L-glucose, suggesting that glucose-derived moieties contribute to cellular internalization. Although this effect does not directly identify a specific transporter, it supports the concept that glucose-functionalized nanoparticles exhibit enhanced intestinal engagement compared with plain nanoparticles.

The improved EPA and DHA deposition observed in the present study aligns with this phenomenon, as TNPs resulted in greater EPA deposition at 4 h post-gavage and higher DHA deposition from 4 h to 24 h. These findings suggest a possible ligand-assisted uptake process at the level of the intestinal microvilli, potentially involving glucose-related interaction pathways. This interpretation is consistent with the known roles of intestinal transporters such as SGLT1 and GLUT2/5/7, which facilitate the uptake of glucose and structurally related monosaccharides^74–76^.

Similarly, several studies have demonstrated that nanoparticle internalization can be enhanced when particles are functionalized with specific monomolecular ligands. For example, ligand-dependent uptake has been observed with nanoparticles conjugated to galactose^77^, L-carnitine^68^, and taurocholic acid^69^, showing increased interaction with intestinal or muscle cells^20^. These examples support the broader concept that surface-presented biological ligands can promote selective engagement with epithelial transport mechanisms, although the precise transporter responsible for APG-modified nanoparticle uptake remains to be clarified.

Nonetheless, the present results do not establish whether GLUTs or other carbohydrate-related transporters directly mediate the enhanced uptake observed with TNPs. Intestinal epithelial cells possess multiple endocytic and transporter-associated pathways capable of internalizing nanoscale materials^78^, and these may differentially contribute to systemic delivery^79^. Future mechanistic studies, including receptor-blocking assays, in vivo glucose-competition experiments, selective transporter inhibition, and characterization of cellular trafficking pathways (such as endosomal and lysosomal processing), will be required to determine the specific routes underlying APG-mediated uptake in chickens.

The coefficient of efficacy in the TNPs groups indicated that the targeting process had potential to transfer the n-3 PUFA into target organ. The transfer efficacy data (Table 6) reinforce this interpretation, as TNPs consistently exhibited higher deposition of both EPA and DHA across all sampling times compared with non-modified NPs. Notably, DHA transfer was markedly elevated in the TNP group from 4 h to 24 h, suggesting that APG functionalization improves intestinal uptake and prolongs systemic availability.

In contrast, the negative or near-zero coefficients observed in the NP group at early time points (e.g., − 1.41% and − 1.85% at 4 h) indicate inefficient absorption and potentially rapid clearance of plain nanoparticles. The slight reduction in n-3 PUFA amounts observed at certain time points may reflect redistribution of fatty acids to other tissues or increased metabolic utilization; however, the present data are insufficient to determine the underlying pathways. Similar processes have been described in nanoparticle systems in other species^67,79–81^. Further studies should therefore examine plasma lipid profiles to characterize the circulation kinetics of lipid nanoparticles in chickens, and haematological and serum biochemical parameters to assess their systemic effects. In addition, identifying the optimal supplementation level of APG-modified nanoparticles is essential, as dose-dependent differences in absorption and tissue deposition may influence both muscle enrichment and the distribution of fatty acids across key organs, thereby providing a more complete understanding of n-3 PUFA delivery dynamics.

## Conclusions

This study demonstrated the successful design and application of glucose transporter-targeted lipid nanoparticles (TNPs) for enhancing the delivery and utilization of n-3 PUFA-rich tuna oil in poultry. The findings highlighted that oil source played a critical role in determining key nanoparticle properties, including particle size, dispersion stability, and zeta potential. Thermal and chemical analyses confirmed the encapsulation of n-3 PUFA oils within the lipid matrix, as evidenced by the reduced melting points observed through DSC analysis. Notably, the nanoencapsulated oils showed improved oxidative stability compared to unencapsulated oils, with degradation rates influenced by storage temperature. Most importantly, the in vivo trial confirmed the effectiveness of targeted delivery in promoting the deposition of long-chain n-3 PUFAs in chicken breast muscle. These results offer new insights into the use of targeted nanotechnology to produce functional poultry meat enriched with health-promoting fatty acids. This approach holds promise for future application in commercial feed systems aimed at improving meat quality and nutritional value in the poultry industry.

## Materials and methods

### Experimental design for in vitro study

For the in vitro study, four preparations were considered, each with three replications. These were compared with free oils (crude tuna oil, TO; feed grade, T. C. Union Agrotech Co. Ltd., Bangkok, Thailand and algal oil, AO (Schizochytrium sp.); Shanghai Wellboost Health Food Co., Ltd, Shanghai, China), and TO and AO loaded non-targeted nanoparticles (NP) and targeted nanoparticles (TNPs), respectively, including TO_NPs, AO_NPs, TO_TNPs and AO_TNPs. The physicochemical characteristics and in vitro storage stability of lipid nanoparticles were investigated.

### Lipid nanoparticles synthesis

**Fig. 4:**
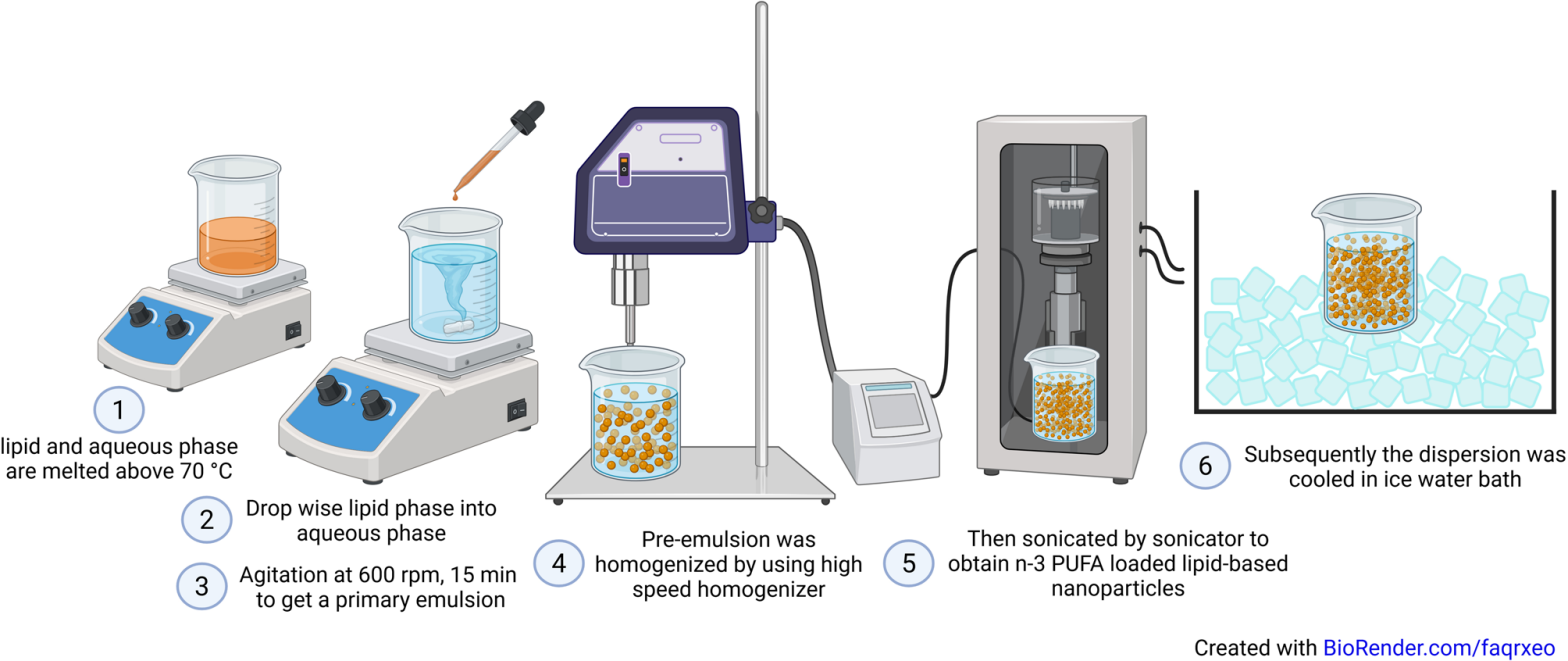
Lipid nanoparticle preparation. Created in BioRender. Homyok, P. (2025) https://BioRender.com/faqrxeo.

The n-3 PUFA loaded lipid-based nanoparticles were prepared by hot and high-pressure homogenization approach (Fig. 4) as previously described^82^. In this method, the lipid phase consisting of GMS (5 g), span 80 (3 g) (Croda, Bangkok, Thailand) and n-3 PUFAs (20 g) (tuna oil or algal oil) was blended and melted above 70 °C to form an organic phase. The fatty acid composition of crude tuna oil and algal oil is indicated on Table 7. Meanwhile, the aqueous phase was prepared by dispersing emulsifying agent, glycerol (2.5 g) Tween 20 (3 g), Poloxamer 188 (2 g) (Croda, Bangkok, Thailand) in distilled water. The aqueous phase added drop wise to lipid phase at above 70 °C with continuous agitation at 300 rpm for 3 min to get a primary emulsion. The pre-emulsion was then homogenized by using high-speed homogenizer (IKA, Altra-Turrac T25, Germany) at 8,000 rpm for 3 min. Then, it was sonicated (Qsonica sonicator, USA) at 30 A pulse on 30 s and off 10 s intervals for 5 min to obtain n-3 PUFA loaded non-targeted lipid-based nanoparticles (NPs). For targeted lipid-based nanoparticles (TNPs), 2 g of alkyl polyglucoside was blended with organic phase preparation. Subsequently the dispersion was cooled in the ice water bath. The prepared dispersion was stored in airtight container at 4 °C. A schematic diagram was created using BioRender (BioRender.com, version current as of 2025).

The encapsulation efficiency (EE%) and loading capacity (LC%) of total tuna oil (as shown Table 7) were calculated for the tuna oil-loaded nanoparticles using the following formulas:

$$\:\mathrm{E}\mathrm{E}\mathrm{\%}\:=\:\left(\frac{{W}_{\mathrm{e}\mathrm{n}\mathrm{c}\mathrm{a}\mathrm{p}\mathrm{s}\mathrm{u}\mathrm{l}\mathrm{a}\mathrm{t}\mathrm{e}\mathrm{d}}}{{W}_{\mathrm{i}\mathrm{n}\mathrm{i}\mathrm{t}\mathrm{i}\mathrm{a}\mathrm{l}}}\right)\times\:100,\:\mathrm{L}\mathrm{C}\mathrm{\%}\:=\:\left(\frac{{W}_{\mathrm{e}\mathrm{n}\mathrm{c}\mathrm{a}\mathrm{p}\mathrm{s}\mathrm{u}\mathrm{l}\mathrm{a}\mathrm{t}\mathrm{e}\mathrm{d}}}{{W}_{\mathrm{l}\mathrm{i}\mathrm{p}\mathrm{i}\mathrm{d}\:\mathrm{t}\mathrm{o}\mathrm{t}\mathrm{a}\mathrm{l}}}\right)\times\:100$$

where W_initial_ is the amount of total tuna oil initially added to the system, W_encapsulated_ is the amount retained in the final nanoparticle matrix, and W_lipid total_ is the total lipid used for nanoparticle synthesis. To determine the amount of encapsulated lipid (W_encapsulated_), the nanoparticle suspension was first centrifuged to separate and remove the unencapsulated oil. The resulting nanoparticle pellet was then subjected to lipid extraction using chloroform: methanol (2:1, v/v), following a modified Folch procedure to release the entrapped lipid from the nanoparticle matrix. The recovered lipid was subsequently converted into fatty acid methyl esters (FAMEs) and analyzed by GC–MS, as described in the Fatty acid composition section. The quantified fatty acids obtained from this extraction were used as W_encapsulated_ for the EE% and LC% calculations.

**Table 7:** Fatty acid composition of n-3 PUFA oils (% of total fatty acids).

Fatty acid composition	TO	AO	TO loaded in nanoparticles
Palmitic Acid (C16:0)	20.28	38.01	29.43
Stearic Acid (C18:0)	5.28	1.47	8.46
Oleic Acid (C18:1n9)	14.16	-	22.64
Linoleic Acid (C18:2n6)	2.84	0.23	7.69
α-Linolenic acid (C18:3n3)	1.68	0.59	2.40
Arachidonic Acid (C20:4n6)	2.52	0.49	4.30
Eicosapentaenoic acid (C20:5n3)	6.98	0.72	3.60
Docosahexaenoic acid (C22:6n3)	20.03	37.63	9.24
SFA	39.15	52.91	43.95
MUFA	24.39	-	28.82
PUFA	36.45	46.96	27.23
n-3 PUFA	30.30	46.01	15.24
n-6 PUFA	6.11	8.02	11.99
n-6/n-3 ratio	0.20	0.17	0.79
Encapsulation efficiency (EE%)	-	-	79.23%
Loading capacity (LC%)	-	-	52.82%

*SFA* Saturated fatty acid, *MUFA* Monounsaturated fatty acid, *PUFA* Polyunsaturated fatty acid, *n-3 PUFA* Omega-3 polyunsaturated fatty acids, *n-6 PUFA* Omega-6 polyunsaturated fatty acids. *TO* tuna, *AO* algal.

### Physicochemical characteristic analysis

The particle size, polydispersity index (PDI), and zeta potential of the lipid nanoparticles (NPs and TNPs) were measured using a Zetasizer 3000HS (Malvern Instruments, Malvern, UK) based on dynamic light scattering (DLS) at 25 °C. Samples were appropriately diluted with deionized water prior to measurement to avoid multiple scattering effects. Each measurement was performed in triplicate, and results were expressed as mean ± SE.

### Thermal and chemical properties of lipid nanoparticles

#### Differential scanning calorimetry (DSC)

DSC is a thermo-analytical technique in which the difference in the amount for heat required to increase the temperature of a sample and reference is measured as a function of temperature. Usually, both the sample and reference are maintained at nearly the same temperature throughout the experiment. Generally, the temperature program for a DSC analysis is designed such that the sample holder temperature increases linearly as a function of time. The reference sample should have a well-defined heat capacity over the range of temperatures to be scanned. DSC (Pyris I DSC, Perkin Elmer, USA) was performed on samples of nanoparticles with and without feed or oil ingredient components to determine whether there were any interactions with the core-shell components.

To determine the phase transition temperatures of the nanoparticles content n-3 PUFA and GMS, dried samples were placed in open aluminum pans. An empty pan was used as a reference pan compared with sample pan. Baseline correction and data treatment were conducted with OriginPro 9.85 (OriginLab, Northampton, Massachusetts, USA).

#### Fourier-transformed infrared spectroscopy (FTIR)

In order to evaluate potential interactions between functionalized ligands and excipients within the lipid nanoparticles, a FTIR spectrum was acquired. The samples, consisting of solid lipid, liquid lipid, glucose-functionalized ligand, surfactant, physical mixture, non-target lipid nanoparticles loaded with tuna oil and algal oil, as well as targeting lipid nanoparticles loaded with tuna oil and algal oil, were meticulously ground with potassium bromide (KBr) to generate an infrared transparent matrix. Subsequently, FTIR scanning was performed using a Shimadzu Corporation FTIR-8400 S spectrometer, covering the spectral range of 4,000 to 500 cm^− 1^ with a resolution of 1.0 cm^− 1^. Baseline correction and data treatment were conducted with OriginPro 9.85.

### In vitro storage stability

The assessment of storage stability was conducted following the methodology outlined as previously described^82^ with some modifications. Free oil and oil synthesized within nanoparticles (*n* = 3) were stored at 4 °C in stability cabinets during four weeks. The hydrodynamic diameter (HD) was measured at different time points by dynamic light scattering (DLS) using a Malvern Zetasizer at an angle of 90° at 25 °C and the oxidative stability was investigated by measuring PV and TBARS value.

### In vitro oxidative stability and peroxide value

The determination of the primary oxidation products was carried out using the procedure outlined as previously described^83^. In order to measure the PV, the sample was added to 9.8 mL of a chloroform: methanol mixture (2:1, v/v) and mixed for 5 s on a vortex mixer. Subsequently, a solution of ammonium thiocyanate (50 µL) was introduced into the mixture, followed by the addition of iron (II) solution (50 µL). The iron (II) solution was prepared by combining BaCl_2_ and FeSO_4_ at a final concentration of 0.144 mol L^− 1^, resulting in individual concentrations of 0.132, respectively. After 5 min incubation period at room temperature, the absorbance of the samples was measured at 500 nm against a blank (solution without any sample) by a Shimadzu-1800 UV–visible spectrophotometer (Shimadzu, Tokyo, Japan). The quantities of lipid hydroperoxides were determined by employing an external standard curve constructed with cumene hydroperoxide (purity 80%, sigma-Aldrich Co, USA) with concentrations ranging between 0 and 16 µM.

### In vitro oxidative stability and thiobarbituric acid reactive substances

The secondary oxidation products were monitored by quantification of the thiobarbituric acid (TBA) reactive substances (TBARS) as previously described^84^. In brief, the samples were mixed with 1.8 mL of deionized water and 4.0 mL of TBA solution. TBA solution was prepared by dissolving 15 g of trichloroacetic acid (15% w/v) and 0.375 g of TBA (0.375%) in 100 mL of 0.25 mol L^− 1^ HCl. In the following, the mixtures were heated in a boiling water bath for 15 min and then cooled to room temperature. Finally, the mixtures were centrifuged (2000 g for 15 min). The intensity of the color created as a result of the reaction between TBA with malondialdehyde (MDA), an important by-product of lipid peroxidation, was measured at 532 nm. The standard curve of 1,1,3,3-tetraethoxypropane was used to determine the MDA concentrations.

### Experimental design for in vivo study

All procedures in the present study were approved by the Ethics Committee on Animal Use of the Suranaree University of technology, Nakhon Ratchasima, Thailand (SUT-IACUC-009/2021). The experiment was conducted at Suranaree University of Technology (SUT) farm. The experimental model employed a completely randomized design, comprising three treatments and three replicates. The treatments consisted of control, tuna oil-loaded non-targeted lipid nanoparticles and tuna oil-loaded targeted lipid nanoparticles. Thirty-three female chickens, aged 56 days with an average weight ranging from 750 to 800 g, were individually housed in cages for a period of 7 days as an adaptation phase prior beginning the experiment. The chickens received commercial diet and water ad libitum for the whole period and withdrawal feeding 12 h before investigation. At 63 days of age, the lipid nanoparticles (2 ml for each chicken) were administered through oral administration at once, utilizing a syringe affixed to a designated feeding tube. After administration, the chickens were euthanized by cervical dislocation at distinct time intervals after gavage, 2, 4, 8, 12, and 24 h (3 chickens per treatment and per time interval). At each time point, all the breast muscle of the chickens was harvested and stored at -25 °C for imaging purposes, as well as for the subsequent analysis of fatty acid composition.

### In vivo imaging of tissue biodistribution

The investigation of nanoparticle distribution in muscle samples involved assessing the quantity of nanoparticles deposited in the target organ by utilizing the optical in vivo imaging system (IVIS), which captured the signal emitted by the fluorescent molecules. The fluorescence was observed within the appropriate wavelength range during the absorption (excitation) and emission processes. This methodology bears resemblance to a confocal laser-scanning microscope. However, the IVIS system is specifically designed for detecting signals from larger specimens. In this study, this instrument was employed to validate the presence of lipid nanoparticles within the target organ, the muscle.

The In vivo imaging of nanoparticle accumulation in birds was assessed following the methodology as previously described^14^. The birds were administered NR-NPs or NR-Glu-NPs orally at a dose of 1 mg/kg (equivalent to 3.14 µM, 2 mL) via oral gavage. Samples were eliminating unwanted fluid before quantifying the near-infrared fluorescence signal intensities by using an In Vivo Imaging System MS FX PRO (Carestream Health Inc., Rochester, NY, USA) with an excitation band pass filter at 540 nm and an emission at 600 nm. Images were processed using Bruker Molecular Imaging software version 7.1.3.20550 (Bruker, Billerica, MA, USA). Mean intensity was performed to analyze statistics. The Nile red concentration was equivalent in all experiments (*n* = 3 birds per treatment).

### Fatty acid composition

The lipids were extracted from approximately 5 g of each muscle sample using 90 ml of chloroform: methanol (2:1, v/v)^85^. After that, the methylation was conducted with around 20 to 25 mg of extracted fat^86^. The fatty acid methyl esters (FAME) were analyzed using gas chromatography (Hewlett-Packard 7890 A; Agilent Technologies, Santa Clara, CA, USA) with a capillary column (SP 2560, Supelco Inc., Bellefonte, PA, USA, 100 m × 0.25 mm i.d., 0.20-µm film thickness) and a flame ionization detector. The carrier gas was helium at a flow rate of 0.95 ml/min. The temperatures of the injector and detector were 260 °C. The initial column temperature was 70 °C. It raised to 175 °C at a rate of 13 °C/min, and then to 240 °C at a rate of 4 °C/min. Compound were identified and quantified by using Masshunter software (v10.0.707.0, Agilent, USA). Coefficient of efficacy (%) was performed to evaluate the potential of lipid nanoparticles using the following formula:

$$Coefficient{\text{ }}of{\text{ }}efficacy{\text{ }}\left( \% \right)=\left( {\frac{{FAn~in~sample~treated~ - ~FAn~in~untreated}}{{FAn~amount~in~suspensions~used~}}} \right) \times 100$$

### Statistical analysis

Analysis of variance will be performed by GLM procedure for a completely randomized design using SPSS Version 18.0 (SPSS Inc., Chicago, I11., USA). University Edition with the following statistical model:

$$Yij{\text{ }}={\text{ }}\mu {\text{ }}+{\text{ }}\tau i{\text{ }}+{\text{ }}\varepsilon ij$$

Where: Yij = the dependent variable, µ = the overall mean, τi = the treatment effect, and εij = the random residual error. Significant differences between treatment means were assessed by Tukey’s multiple comparison tests after a significant F-test. The level of statistically significance will establish at *P* < 0.05. The values for physical characteristic of lipid nanoparticles were expressed as means ± standard error (SE) and the value for storage stability and fatty acid composition were expressed as means ± standard error of mean (SEM), which represents the pooled SEM for the model.

## Data Availability

The datasets generated during and/or analyzed during the current study are available from the corresponding author on reasonable request.

## Declarations

### ARRIVE guidelines statement

This study is reported in accordance with ARRIVE guidelines (https://arriveguidelines.org).
